# Delayed onset phlegmasia cerulea dolens post-SARS-CoV-2 infection treated with minimally invasive clot retrieval technology

**DOI:** 10.1016/j.jvscit.2022.11.019

**Published:** 2022-12-17

**Authors:** Michael Carey, Osaid Alser, Jessica Leist, Kelsee Sorrells, Brittany K. Bankhead, Wei Li

**Affiliations:** a School of Medicine, Texas Tech University Health Sciences Center, Lubbock, TX; b Department of Surgery, Texas Tech University Health Sciences Center, Lubbock, TX; c Surgical ICU Services, University Medical Center, Lubbock, TX

**Keywords:** COVID-19, Phlegmasia cerulean dolens, minimally invasive percutaneous mechanical thrombectomy

## Abstract

Coronavirus disease 2019 is associated with a significant venous thromboembolic risk. Phlegmasia cerulean dolens is a severe form of deep vein thrombosis that can lead to acute limb ischemia. In this report, we present a 58-year-old woman who developed a delayed-onset left lower extremity phlegmasia cerulean dolens 8 weeks after coronavirus disease 2019 onset that led to compartment syndrome and acute limb ischemia from external compression of the arterial vasculature from edematous muscle. The patient received an emergent minimally invasive percutaneous mechanical thrombectomy and four-compartment fasciotomy, resulting in adequate perfusion and ultimately made a full recovery.

Phlegmasia cerulean dolens (PCD) arises from the acute complete or near-complete occlusion of the iliofemoral venous outflow system by thrombosis, which leads to rapidly developing interstitial edema, high compartment pressure from external compression of the arterial vasculature by edematous muscle, and eventually acute lower extremity ischemia.^1^^,^^2^ Coronavirus disease 2019 (COVID-19) (which is caused by severe acute respiratory syndrome coronavirus 2 [SARS-CoV-2] infection) has been shown to induce a hypercoagulable state through endothelial injury, stasis, and the induction of prothrombotic factors.^3^, ^4^, ^5^, ^6^ Recent reports have showed association between SARS-CoV-2 infection and PCD owing to a hypercoagulable state; however, no reports have shown an association with COVID-19 and PCD beyond 3 weeks of COVID-19 onset.^7^, ^8^, ^9^ This article describes a case of delayed-onset PCD that led to compartment syndrome and acute limb ischemia, which was treated successfully with an emergent minimally invasive percutaneous mechanical thrombectomy and four-compartment fasciotomy. The institutional review board provided an exemption, and the patient consented to publishing case details and images.

## Case presentation

A 58-year-old woman with medical history significant for chronic obstructive pulmonary disease and heavy cigarette smoking (40 pack-year, quitting 2 months before presentation to us), developed COVID-19 pneumonia that required 6 weeks of hospitalization at an out-of-state hospital. The patient relied heavily on oxygen during that admission and was discharged from that outside hospital with a new baseline oxygen requirement of 3 to 4 L. The patient was not vaccinated against SARS-CoV-2. Two weeks after the above hospital discharge, the patient presented to the referring facility’s emergency department with an acute onset of intermittent left lower limb numbness, pain, and coldness after doing some routine work in her garden. The patient reported no family history of deep vein thrombosis (DVT) or hypercoagulable disorders. On physical examination, the patient had edema and ecchymosis up to the mid-calf, in addition to absent dorsalis pedis and posterior tibial pulses and Doppler signals in the left lower extremity (LLE). A computed tomography (CT) arteriogram (not viewable initially upon the arrival of transfer) at the outside facility indicated massive left leg swelling with edema of the muscles of the distal thigh downward, as well as acute occlusion of all arteries distal to the left popliteal artery. Despite that, the patient was placed on a heparin drip and transferred to a tertiary (our) facility 11 hours after her symptoms began, the patient’s symptoms seemed to progress with persistent numbness and further coldness of her LLE.

**Fig 1:**
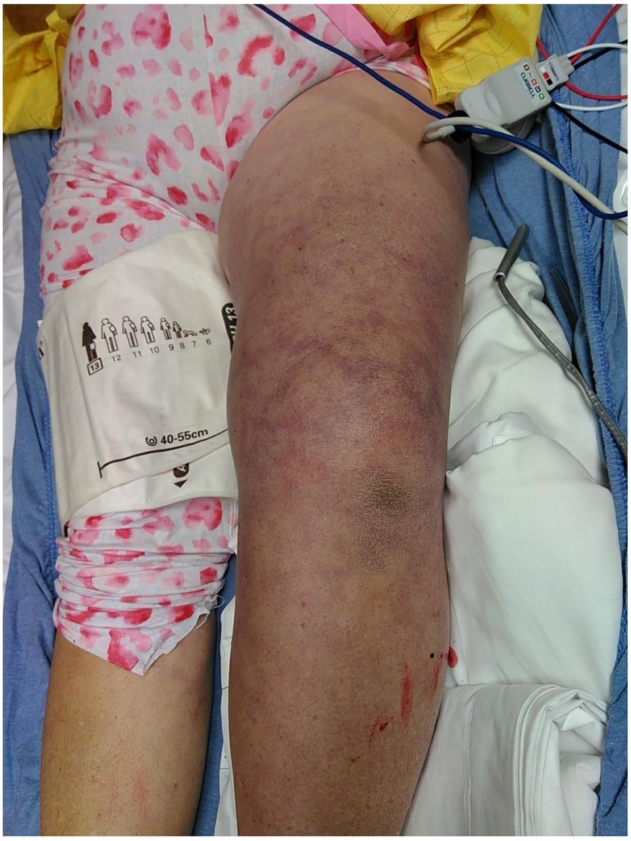
Mottled appearance of massively swelling left leg (preoperatively) consistent with phelgmasia cerulea dolens upon the arrival of transfer.

**Fig 2:**
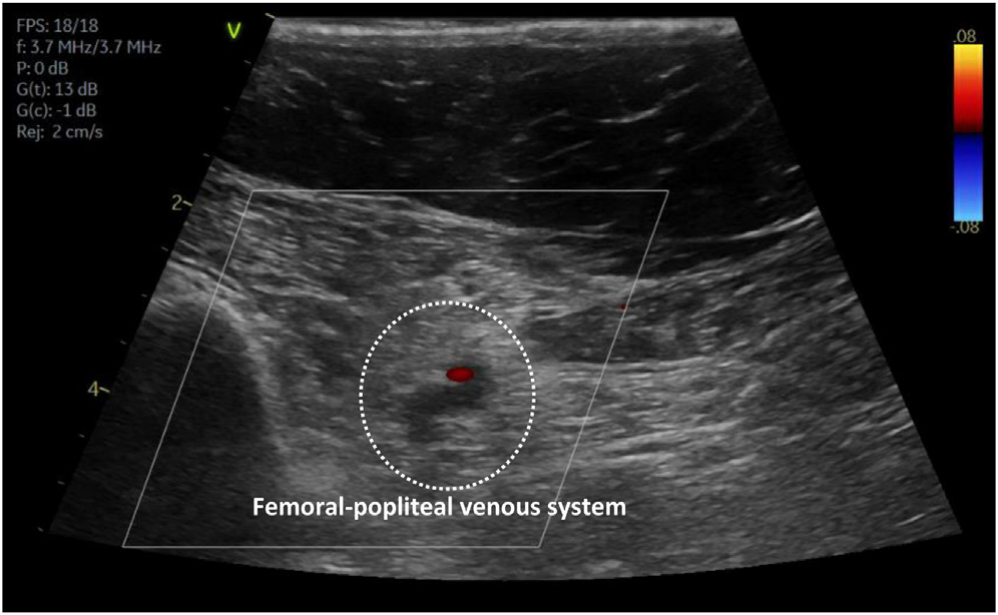
Representative venous duplex of the left femoropopliteal venous system showing clot burden and decreased flow in deep vein thrombosis (DVT).

**Fig 3:**
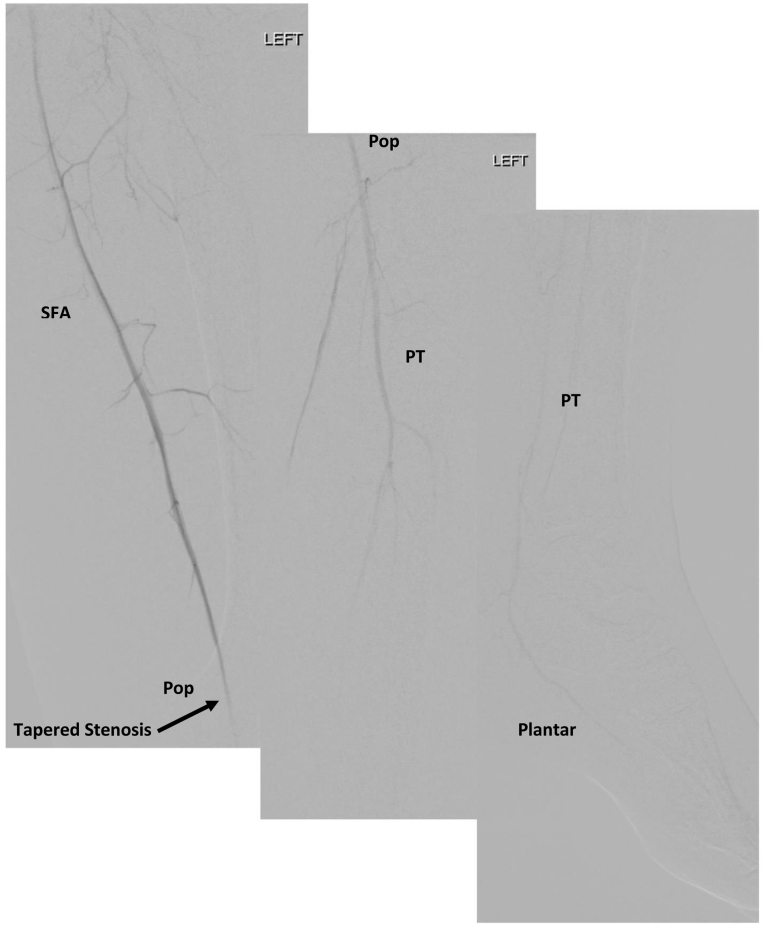
Left leg arterial angiogram indicating a tapered stenosis distal to the proximal popliteal artery (*Pop*), consistent with external compression from compartment syndrome and phelgmasia cerulea dolens (PCD). *PT*, posterior tibial artery; *SFA*, superficial femoral artery.

**Fig 4:**
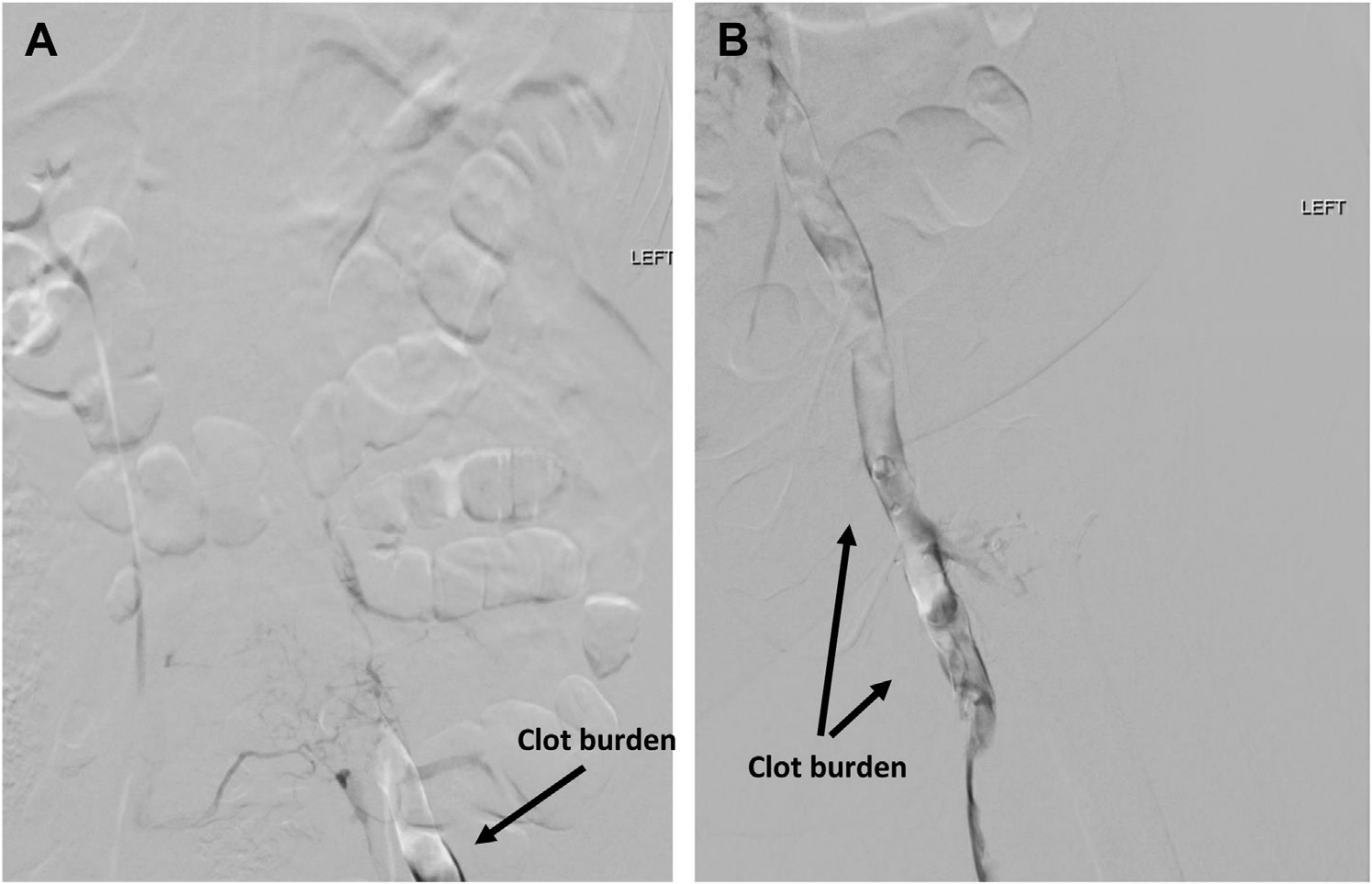
Initial venogram images with contrast injection indicating extensive iliofemoral **(A)** and popliteal **(B)** DVT with labelled clot burden.

**Fig 5:**
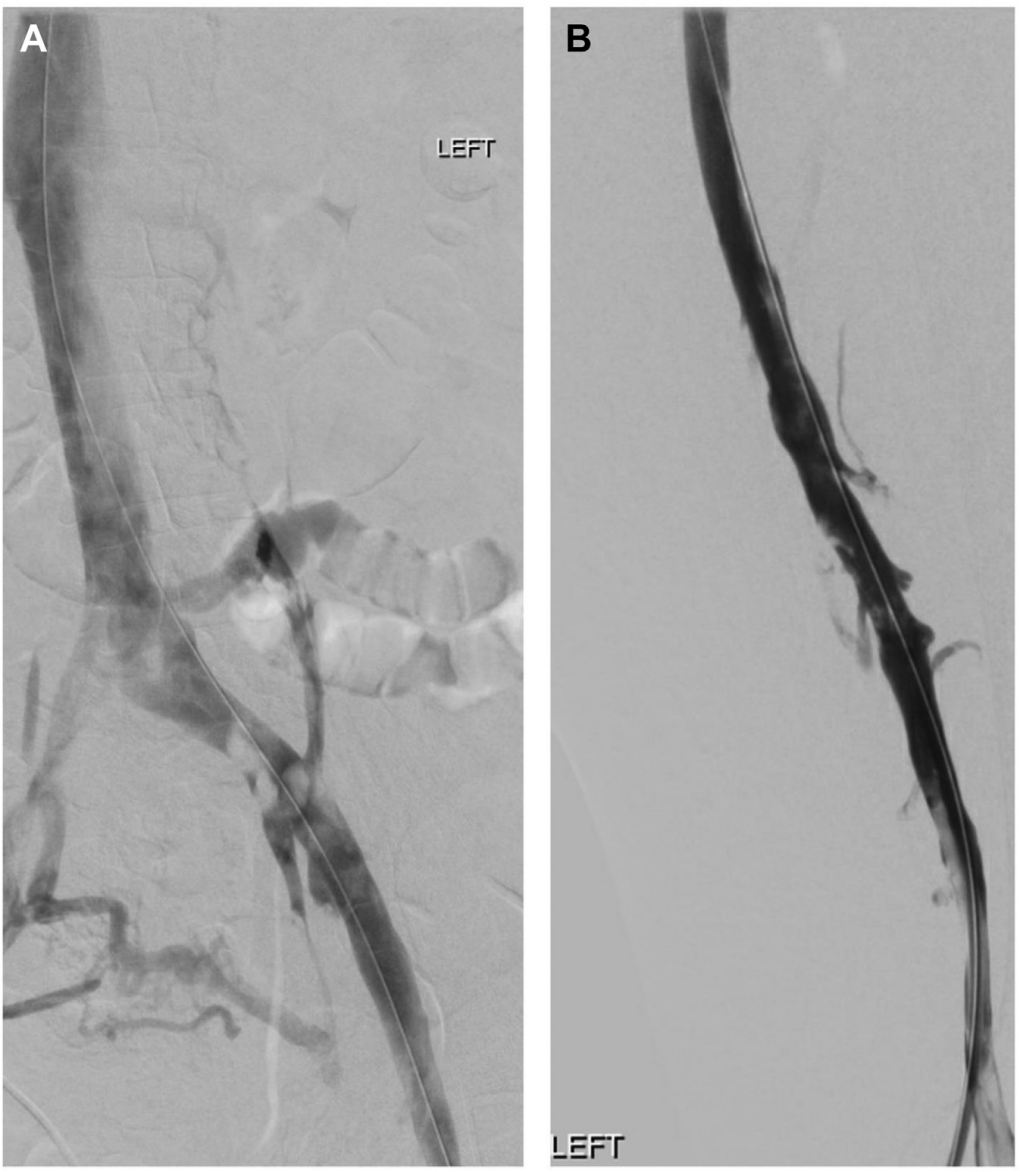
Venogram showing gross resolutions of left iliofemoral **(A)** and femoropopliteal popliteal **(B)** DVTs after Clot-Triever mechanical thrombectomy.

Upon arrival to the new facility, physical examination showed a cold LLE that was mottled and edematous from the mid-thigh downward (Fig 1) with persistent decreased sensation to light touch and absent pulses (left popliteal and distally) both on palpation and on Doppler examination. The patient was emergently brought into the operating room (OR). An on-the-OR table LLE duplex examination was performed and showed severe acute DVT in the left femoropopliteal venous system (Fig 2). The patient was diagnosed with LLE PCD. Although there was a previous CT angiogram that reported acute arterial occlusion at the left popliteal artery, the time of contrast administration was suboptimal. Under general anesthesia, a conventional arteriogram (Fig 3) through the left common femoral artery was carried out in the OR and showed patency of the left common femoral, proximal profunda, superficial femoral system, and tapered stenosis of the popliteal artery. Tapered stenosis of the lower extremity vasculature is often the typical presentation of external compression of arteries by edematous muscle in compartment syndrome, which correlated clinically with the patient’s absent pedal pulses, edematous muscle, numbness, and pain. Distally, the stenotic posterior tibial artery was the only vessel runoff across the ankle, with the anterior tibial and peroneal arteries being occluded distal to the level of mid-calf. With the same supine position and through a left popliteal venous access, a venogram of the leg showed acute DVT and occlusion of the iliofemoral (Fig 4, A) and high-grade stenosis of femoropopliteal (Fig 4, B) venous systems. An AngioJet chemical-mechanical thrombectomy device (Boston Scientific, Boston, MA) was used in the femoral vein starting at the level of the distal thigh and advanced proximally to the iliac system, with 6 mg of tissue plasminogen activator delivered via power pulse and an additional 4 mg of tissue plasminogen activator delivered via hand injection. After the chemical-mechanical thrombectomy, several sequential balloon venoplasties were performed in the femoral and iliac vein systems. However, a repeat venogram showed suboptimal clot removal with a significant amount of clot residual in the left iliac vein system. The Clot-Triever catheter system (Inari Medical, Irvine, CA) was subsequently deployed and retrieved a large amount of clot from the left iliac system through three passes. A completion venogram (Fig 5, A and B) showed satisfactory results with direct venous flow from the left popliteal and femoral vein to the inferior cava system. Upon completion of the thrombectomy, the patient’s lower extremity pulses were evaluated on the table with Doppler examination, which showed absent pedal pulses on the LLE still and multiphasic pulses on the contralateral side. Additionally, the patient’s LLE was still edematous with rigidity in the lower leg muscles. Given the clinical presentation of compartment syndrome and expected muscle reperfusion injury, a LLE four-compartment fasciotomy was performed, which relieved the high pressure of the LLE compartments where muscle groups seemed to be viable within. Strong multiphasic Doppler signals were appreciated immediately after the fasciotomy. Hours after the surgery, the patient's leg seemed to be grossly normal color with decreased swelling on postoperative day 0. A hypercoagulability workup found low antithrombin III (53) and elevated factor VIII (>300) and vWF (417). However, hematology deemed factor studies not reliable in this patient’s acute phase and considered this patient’s DVT as likely provoked from recent COVID-19, which was consistent with others’ reports on COVID-19-related hypercoagulopathy.^10^, ^11^, ^12^ In addition to full therapeutic anticoagulation, the patient was not given aspirin or other antiplatelet agents during the hospital admission. On postoperative day 5, the patient’s respiratory status worsened with significant increased respiratory rate and desaturation. Subsequently, the patient required intubation owing to acute respiratory failure mainly deemed to be secondary to a post-COVID-19 lung baseline condition present upon the current admission. A relatively aggressive hydration after the thrombectomy and fasciotomy to treat suspected rhabdomyolysis served as another precipitating factor for the respiratory failure. The patient had a CT angiogram, which ruled out a pulmonary embolism. In addition, the patient had been on therapeutic anticoagulation since admission, which also made a pulmonary embolism a less likely event. The patient’s respiratory status improved after COVID-specific weaning protocols (which were modified from others in the literature) and dirusesis.^12^, ^13^, ^14^, ^15^ The fasciotomy incisions were managed by wet-to-dry dressings followed by wound vac therapy in both the inpatient and outpatient setting. This patient’s fasciotomy incisions healed completely without any functional deficit.

**Fig 6:**
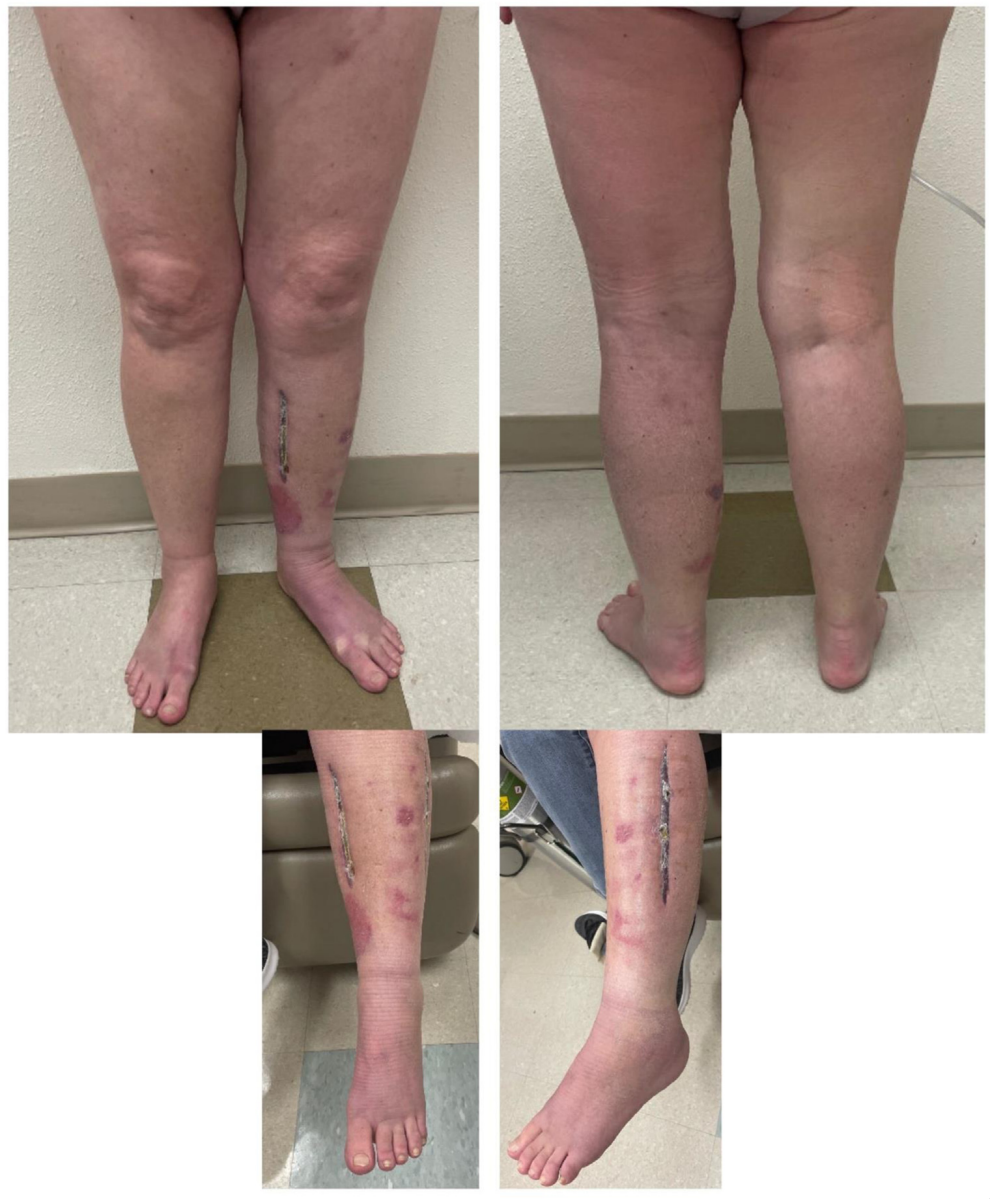
Patient at clinic follow-up 4 weeks after discharge showing well-healing fasciotomy scars and resolution of her phlegmasia cerulean dolens (PCD) with significantly decreased left lower extremity (LLE) edema.

**Fig 7:**
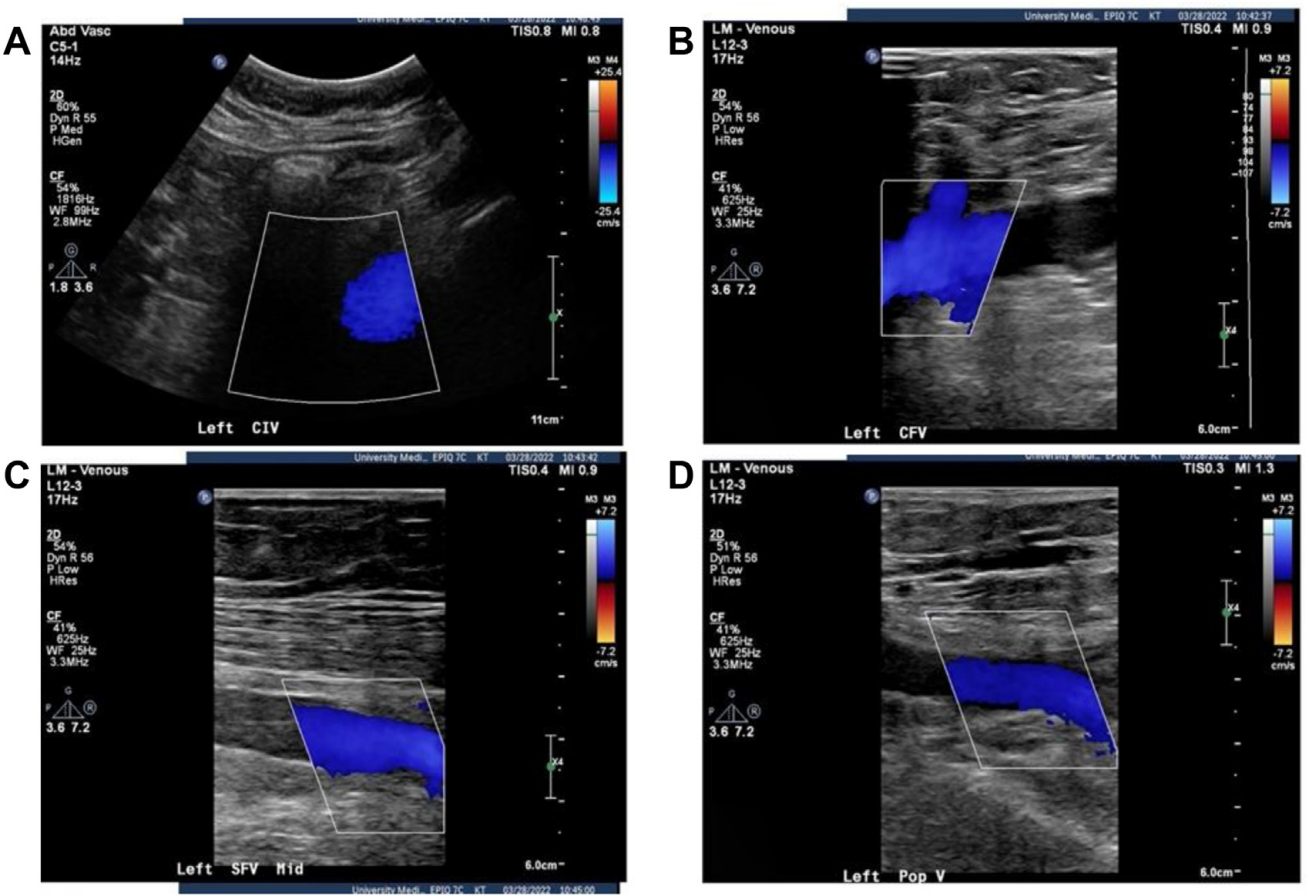
Three-month clinic follow-up with venous duplex indicating normal venous findings without residual superficial vein thrombosis or deep vein thrombosis (DVT) in the left **(A)** common iliac vein, **(B)** common femoral vein, **(C)** superficial femoral vein, and **(D)** popliteal vein.

**Fig 8:**
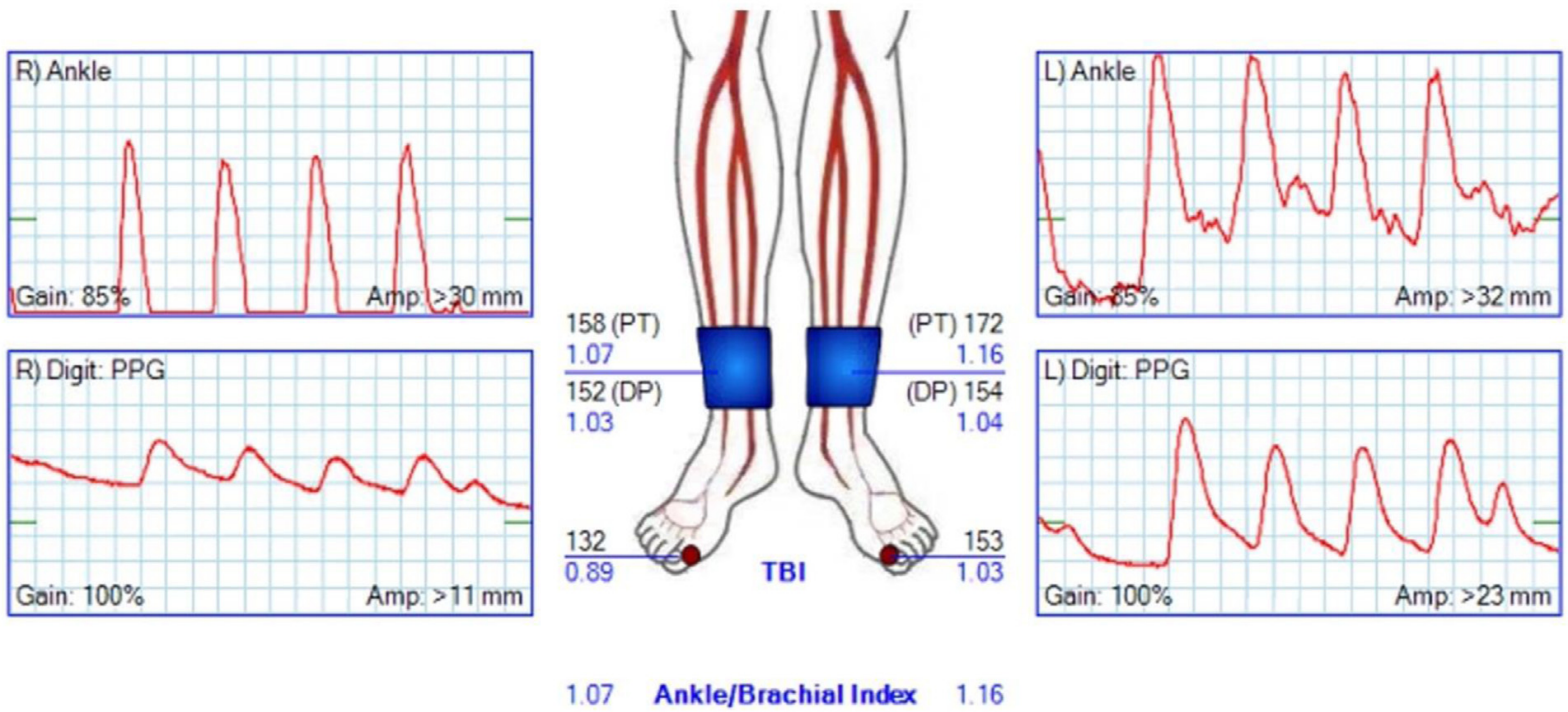
Three-month clinic follow-up with noninvasive arterial study showing a normal left ankle-brachial index and waveforms. *TBI*, toe-brachial index.

The patient also subsequently resumed normal ambulation and was ultimately discharged to an inpatient rehabilitation facility with short-term anticoagulation (apixaban 5 mg twice daily) for 3 months, as suggested in the literature.^16^ The patient was seen in the clinic 4 weeks after discharge with full resolution of her PCD and good healing of her left leg fasciotomy wound (Fig 6). Three months after discharge, the patient returned to full capacity of living independently with normal venous duplex studies (Fig 7) that showed no evidence of residual DVT or superficial vein thrombosis, and a normal ankle-brachial index (Fig 8) with palpable pulses bilaterally in the feet.

## Discussion

PCD is a severe form of DVT that results from a massive iliofemoral DVT that causes venous outflow obstruction and an increase in tissue pressure, leading to compartment syndrome and eventually acute limb ischemia.^17^ COVID-19 has been shown to induce a hypercoagulable state leading to DVT and PCD through endothelial injury, stasis, and disorders of prothrombotic factors.^3^, ^4^, ^5^, ^6^^,^^11^ Damage to the endothelium leads to an increased release of tissue factor, activating the coagulation cascade many times over, and von Willebrand factor, activating platelet aggregation.^3^ Additionally, proinflammatory cytokines such as IL-1, IL-6, and tumor necrosis factor-α, while routinely released from the endothelium in a controlled fashion, are released in elevated quantities following endothelial disruption, and the perturbation of their delicate balance leads to self-amplification, commonly referred to as a cytokine storm.^3^, ^4^, ^5^ The disproportionate elevation in cytokines leads to an acute phase response that favors a prothrombotic state while inhibiting antithrombotic products, creating an overall environment favorable for thrombus formation.^3^ The combined effects of these processes lead to a complete coagulopathy and conditions favorable to both arterial and venous thrombosis, with the incidence of venous thromboembolism being between 20% and 35% for COVID-19 patients in the intensive care unit.^4^ Thus, anticoagulants have been used in COVID-19 patients for antithrombotic prophylaxis and treatment.^5^^,^^6^ Additionally, the literature supports prophylactic anticoagulation with apixaban for patients discharged after COVID-19 infection.^16^ COVID-19-related hypercoagulopathy has been described by others.^10^, ^11^, ^12^ However, the delayed COVID-19-related DVT was uncommon in literature.^18^, ^19^, ^20^ In addition, a PCD in such a setting has not been recognized. There are no cases demonstrating an association between PCD and COVID-19 beyond 3 weeks after COVID-19 onset.^7^, ^8^, ^9^^,^^21^, ^22^, ^23^

Although the patient’s smoking history may have increased her DVT risk despite her smoking cessation 2 months before presentation, the authors believe the patient’s recent COVID-19 pneumonia was the provoking event for her DVT; there were no other associated comorbidities immediately before her PCD. The patient reported no history of claudication or known atherosclerosis before admission. She also denied any family history of coagulopathies. Additionally, her laboratory results did not conclusively show evidence of a coagulopathy. Therefore, we have no other reason but to believe COVID-19 had contributed to the development of the patient’s PCD, because the patient was unvaccinated against SARS-CoV-2 and experienced a severe course of COVID-19.

Although PCD is not rare, PCD in the setting of such significant venous congestion and edematous muscle that it compresses the arterial vasculature and leads to acute arterial ischemia requiring a fasciotomy is uncommon. The typical treatment for PCD often consists of intravenous heparin and an emergent surgical venous thrombectomy, which relieves the venous congestion and relieves lower extremity edema.^17^^,^^24^ Such traditional surgical venous thrombectomy is an invasive open approach that carries complications, such as groin wound infections.^8^^,^^17^ The recent development of percutaneous thrombectomy devices such as the AngioJet and Clot-Triever now provide a state-of-the-art minimally invasive approach. The Clot-Triever system has been used in several COVID-19 patients with documented PCD; however, those patients ultimately needed amputations.^8^^,^^25^ Our patient’s thrombus burden was successfully decreased with the use of both the AngioJet and Clot-Triever thrombectomy systems with their synergy effects when used in combination.

Fasciotomy is generally deemed unnecessary in the setting of PCD, because the primary issue is a blockage of the venous outflow and recanalization of the deep venous system alone often resolves the condition.^26^ However, the patient we present had signs of arterial ischemia and compartment syndrome in the setting of PCD, such as a history of acute-onset LLE pain, numbness, and coldness, as well as absent pedal pulses when she was normotensive (104/76 mm Hg) and palpable pulses in the contralateral foot upon her presentation in the emergency department. Additionally, the patient’s initial CT scan showed edematous muscle, and her arteriogram showed tapered stenosis of the left popliteal artery, commonly seen in external compression of the artery secondary to edematous muscle in compartment syndrome. The measurement of compartment pressures has shown utility in the diagnosis of compartment syndrome in PCD, with elevated pressures in the range of 40 to 50 mm Hg.^27^ However, compartment pressures were not obtained for this patient. Thus, when pedal pulses were still absent on the table after the completion of the thrombectomy, the decision was made to perform a four-compartment fasciotomy based on this patient's clinical picture. The immediate appreciation of multiphasic Doppler signals in the dorsalis pedis and posterior tibial arteries of the left extremity after the procedure suggested the fasciotomy was therapeutic. The present COVID-19 PCD patient has reminded clinicians that, although fasciotomy is a rarity in PCD management, the prompt recognition and treatment of acute compartment syndrome in PCD is critical for limb salvage and functional recovery.

## Conclusions

PCD is a possible complication of COVID-19 and may present in a delayed fashion, as in our case, indicating a prolonged thromboembolic phase. Although rare, PCD can lead to compartment syndrome and acute limb ischemia because of external compression of the vasculature by edematous muscle, as in the case of this patient. The use of percutaneous thrombectomy technology, such as combined AngioJet and Clot-Triever devices, is safe and effective in removing the clot burden in PCD; however, additional measures may be needed to restore circulation if compartment syndrome develops. A fasciotomy may be required, as in this patient, to prevent further limb ischemia and restore adequate limb perfusion in patients with PCD with compartment syndrome. This case highlights the importance of monitoring a patient’s vascular status in the setting of COVID-19 or PCD and promptly intervening if necessary. Last, further studies are needed to show optimal duration of anticoagulation after PCD after COVID-19.
